# Senolytics in a Model of Alzheimer's Disease

**DOI:** 10.1093/geroni/igab046.2420

**Published:** 2021-12-17

**Authors:** Ellen Wang, Suckwon Lee

**Affiliations:** Buck Institute for Research on Aging, Novato, California, United States

## Abstract

The therapeutic effects of senescent cell killing with senolytics in neurodegeneration mouse models poise this strategy as an intervention candidate for Alzheimer’s Disease (AD). However, it is unclear whether senolytic therapies for AD are translatable to human cells. To determine whether senolytics could be a viable therapeutic for AD, we have treated long-term mixed human neuron/astrocyte primary cultures with amyloid beta oligomers (ABO), which we have shown to induce a phenotype consistent with senescence in neurons. Fifteen days after ABO treatment, we administered Navitoclax (Nav) and the natural killer cell-line NK92, which are known to selectively kill senescent cells in the periphery. Following treatment, we assessed senescence markers in our cultures as well as senescent cell killing selectivity through cleaved Caspase 3 quantification. Our preliminary data show that Nav (8, 4, and 0.5uM) kills both control and ABO treated cells. NK92 cells (10 to 1 effector to target ratio) also kill some control cells, suggesting there is not a clear cut mechanism by which NK92 cells can distinguish senescent from non-senescent neurons or astrocytes. Although analysis of selective killing is ongoing, off-target killing indicates that we need more refined senolytic strategies to implement their safe human use.

